# Effect of binder and activator composition on the characteristics of alkali-activated slag-based concrete

**DOI:** 10.1038/s41598-024-63214-5

**Published:** 2024-06-12

**Authors:** Mohamed Heshmat, Ismail Amer, Fareed Elgabbas, Mohamed A. Khalaf

**Affiliations:** Structural Engineering Department, Faculty of Engineering, Ain Shams University, Cairo, Egypt

**Keywords:** Slag, Lime, Alkali activated, Concrete, Activator, Binder, Compressive strength, Workability, Civil engineering, Composites

## Abstract

Alkali Activated Slag Concrete (AASC) has been a sustained research activity over the past two decades. Its promising characteristics and being environmentally friendly compared to Ordinary Portland Cement made AASC of exceptional interest. However, there is still no firm mix design, for the AASC, that can provide desirable fresh and hardened properties based on the composition of the binder and activator. This research specifically aims to investigate the affecting parameters on the slump and compressive strength of alkali-activated slag/lime-based concrete and provide a better understanding of the potential reasons for these characteristics. The experimental program consisted of two stages; the first stage studied the effect of different binder and activator compositions, and the second stage studied the water-to-binder ratio and binder content effects on the slump and compressive strength of alkali-activated slag/lime-based concrete. The binder and activator compositions were defined through two main parameters, the hybrid factor (HF = CaO/Si_2_O + Al_2_O_3_) and the solution modulus (Ms = SiO_2_/Na_2_O). The compressive strength, initial slump, and slump loss were measured to evaluate the different mixes and specify the optimum range of compositions. Based on the studied parameters, the effective range to achieve desirable slump and concrete compressive strength is from HF 0.6 up to 0.8 at Ms 1.5, this would achieve a compressive strength of more than 30 MPa and a slump of 100 mm after 90 min.

## Introduction

OPC is one of the most common materials in the construction sector. The production process of Ordinary Portland Cement (OPC) is accompanied by a lot of issues, whether environmental or sustainability of resources issues. Environmental issues such as dust, greenhouse gases emissions as carbon dioxide CO_2_ (production of 1 ton of OPC results in 0.9 tons of CO_2_^1^ and consumption of non-renewable energy resources in the production of OPC^2^ motivate the researchers to focus on finding solutions to reduce or eliminate these issues.

The above-mentioned issues can be somewhat overcome by having a reduction in the usage of OPC in the construction sector. Through finding a replacement criterion for OPC either partially or fully, a sustainable and healthier environment can be achieved. Alkali-Activated Materials (AAMs) are a potential solution for the replacement of OPC. AAMs are materials rich in aluminate and silicate which have proven their viability as a cleaner replacement to make cement-free concrete^2,3^. AAMs such as Fly Ash (FA), Ground Granulated Blast Furnace Slag (GGBFS), Rice Husk Ash (RHA), and Silica Fume (SF) are sustainable materials that offer properties comparable to the OPC^4^. Ground Granulated Blast Furnace Slag (GGBFS) as a binder offers superior properties such as high early strength, and temperature resistance when activated using an alkaline activator to produce Alkali-Activated Slag Concrete (AASC)^5^. GGBFS is a byproduct that results during the steel production process, more than 2.4 million tons of GGBFS are produced annually be China only, and only 20–30% of this amount is used effectively while the other amount is treated as waste materials^6,7^.

GGBFS is rich in aluminate and silicate such as the other AAMs, but additionally, it has a considered amount of Calcium Oxide (CaO). The chemical composition of GGBFS can affect its chemical activity, hydration, and polymerization process. Jin et al. concluded that the reaction performance of GGBFS can be indicated effectively through the ratio of (CaO + MgO)/(SiO_2_ + Al_2_O_3_)^8^ Zhu et al. investigated the effect of adding CaO to GGBFS on the properties such as fluidity, setting time, and compressive strength of the Alkali Activated Slag (AAS) pastes and mortars, the addition of CaO increased the setting time and fluidity, while the highest compressive strength was achieved by 25% replacement by weight^9^. A recent study performed by Amer et al. investigated the effect of adding OPC to GGBFS on the workability and the compressive strength of AAS concrete, the addition of OPC with the GGBFS is not recommended due to the obtained low workability while using GGBFS only achieved the best workability and compressive strength^10^.

The activators have a crucial effect on the polymerization process of slag-based systems. Gebregziabher et al. investigated the early-age reaction kinetics and microstructure of AAS paste when activated using different activator types and concentrations. It was concluded that activating GGBFS with NaOH would lead to a rapid reaction process with a very short setting time at ambient temperature, whereas the activation with Na_2_SiO_3_ would lead to higher setting time and better microstructural composition^11^. Li et al. investigated the effect of mixing activator solutions such as Na_2_SiO_3_, NaOH, and Na_2_CO_3_ to control the setting time by altering the polymerization process; it was concluded that the addition of Na_2_CO_3_ to Na_2_SiO_3_ did not affect the polymerization process, while the addition of NaOH to Na_2_SiO_3_ had a noticeable effect on the polymerization which led to increased setting time^12^. Cao et al. investigated the reaction kinetics of AAS pastes using different activators. It was concluded that increasing the solution modulus, Ms (ratio of SiO_2_/Na_2_O) of the activator, which was prepared by mixing NaOH solution with Na_2_SiO_3_ solution, accelerated the hydration process^13^. Amer et al. investigated the effect of solution modulus (Ms) on the slag–cement concrete fresh and hardened properties; it was concluded that the Ms has a negligible effect on the workability^10^. Previous studies were conducted to obtain a mixture design of AASC by controlling the affecting parameters such as solution modulus, water-to-binder content, aggregates content, and GGBFS content^14,15^. However, the previous studies didn’t account for the specific chemical composition of the binder although it’s a major parameter that would change the behavior of the mixture^16^.

As concluded from the above-mentioned literature, more research is needed to understand the effect of the mix composition on the properties of the AAS concrete, especially on the workability and compressive strength. The main objective of this research is to get the optimum range for the binder and activator compositions to achieve the desired slump and compressive strength.

## Experimental program

### Materials

**Figure 1:**
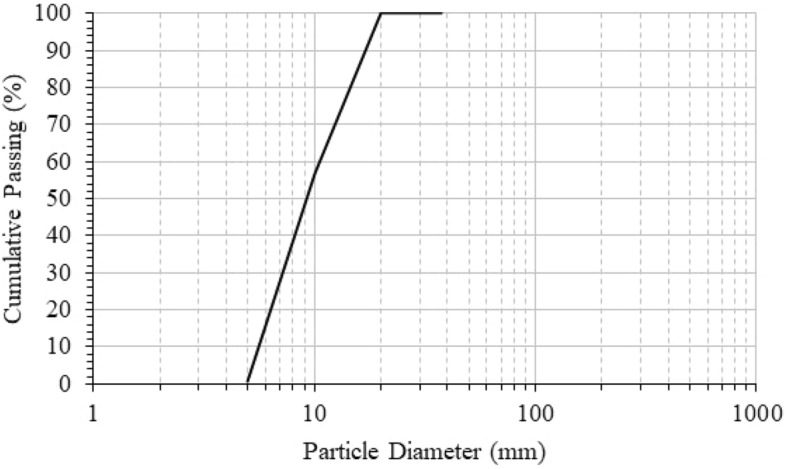
Particle size distribution of the coarse aggregates.

**Figure 2:**
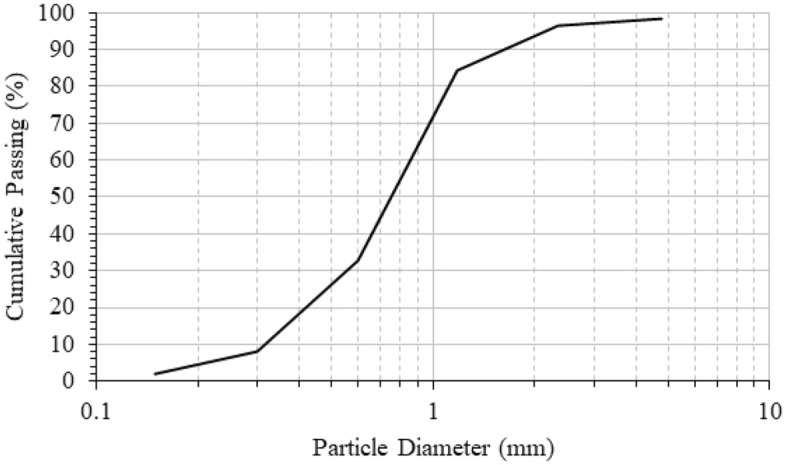
Particle size distribution of the fine aggregates.

In this study, GGBFS and Quicklime (QL) were used as the binder of AASC mixes. The GGBFS has a specific gravity of 2.80 gm/cm^3^ and QL has a specific gravity of 3.34 gm/cm^3^. The chemical composition of both GGBFS and QL was obtained through the XRF test and listed in Table 1. Natural crushed limestone was used as the coarse aggregates with a nominal maximum size of 10 mm and particle size distribution as per Fig. 1, the coarse aggregates satisfy the grading requirements for size number 7 according to ASTM C33/C33M-16, and natural sand was used as the fine aggregates with a fineness modulus of 2.72 and particle size distribution as per Fig. 2, the fine aggregates satisfies the grading requirements according to ASTM C33/C33M-16^17^. To prepare the alkaline activator, Sodium Hydroxide (SH) and Sodium Silicate (SS) were used; a regional commercial producer provided SH as flakes and SS as a liquid. Through the XRF test, the chemical composition of SH and SS was determined and listed in Table 2.

**Table 1:** Chemical composition of used GGBFS and QL (mass%).

Component	SiO_2_	Al_2_O_3_	Fe_2_O_3_	CaO	MgO	K_2_O	Na_2_O	TiO_2_	Mn_2_O_3_	Other oxides	LOI
GGBFS	41.66	13.96	1.49	34.53	5.53	0.97	0.49	0.58	0.35	0.44	–
QL	8.03	2.2	3.18	54.18	1.3	2.76	1.18	0.33	0.06	7.68	19.10

**Table 2:** Chemical composition of used SH and SS (mass%).

Component	SiO_2_	Na_2_O	H_2_O
SH	–	60.25	39.75
SS	31	12	57

### Test matrix

Several parameters related to the chemical composition of both the binder and the activator were studied through different levels for each parameter. The first parameter, that was related to the binder’s chemical composition, was the Hybrid Factor (HF) which is defined as the ratio of CaO/(Si_2_O + Al_2_O_3_) in the binder only rather than the molar ratio. The second parameter, that was related to the activator’s chemical composition, was the Solution Modulus (Ms) which is defined as the ratio of Si_2_O/Na_2_O in the activator. The third parameter was the Binder content. The last parameter was the Water-to-Binder ratio (W/B). Table 3 presents the studied parameters with their different levels. The test matrix was designed based on the traditional factorial method using the parameters and levels mentioned in Table 3; twenty-two mixes were conducted as demonstrated in Table 4. The test matrix is divided into six sets, set 1 to set 4 investigate the variation of HF from 0.6 to 1.2 through Ms from 0.0 to 1.5 while the binder content is constant 400 kg/m^3^ and W/B ratio of 0.50, set 5 investigates the variation of binder content from 450 to 550 kg/m^3^ while the HF is set to 0.6, Ms is set to 1.5, and W/B is set to 0.50, and set 6 investigates the variation of the W/B ratio from 0.40 to 0.55 while the HF is set to 0.6, Ms is set to 1.5, and BC is set to 400 kg/m^3^. The mix proportions were determined for all mixes using the absolute volume approach, this approach states that the volume of all components is equal to 1 m^3^. The mix proportions are illustrated in Table 5. For all mixes, the Na_2_O was constant at 6% by weight from binder content. For all mixes, the extra water content was determined by subtracting the existing water in the SH and SS from the total required water content to achieve the required water-to-binder ratio. The existing water in the SH and SS was determined through the chemical composition of SH and SS that are presented in Table 2.

**Table 3:** The studied parameters and their levels.

HF	Ms	BC	W/B
0.6	0.0	400	0.40
0.8	0.5	450	0.45
1.0	1.0	500	0.50
1.2	1.5	550	0.55

**Table 4:** Test matrix.

Sets	Mix	HF	Ms	BC	W/B
Set (1)	M1	0.6	0.0	400	0.50
	M2	0.6	0.5	400	0.50
	M3	0.6	1.0	400	0.50
	M4	0.6	1.5	400	0.50
Set (2)	M5	0.8	0.0	400	0.50
	M6	0.8	0.5	400	0.50
	M7	0.8	1.0	400	0.50
	M8	0.8	1.5	400	0.50
Set (3)	M9	1.0	0.0	400	0.50
	M10	1.0	0.5	400	0.50
	M11	1.0	1.0	400	0.50
	M12	1.0	1.5	400	0.50
Set (4)	M13	1.2	0.0	400	0.50
	M14	1.2	0.5	400	0.50
	M15	1.2	1.0	400	0.50
	M16	1.2	1.5	400	0.50
Set (5)	M17	0.6	1.5	450	0.50
	M18	0.6	1.5	500	0.50
	M19	0.6	1.5	550	0.50
Set (6)	M20	0.6	1.5	400	0.45
	M21	0.6	1.5	400	0.55
	M22	0.6	1.5	400	0.40

**Table 5:** Mix proportions of All AASC mixes (kg/m^3^).

Mix	GGBFS	QL	SS	SH	Extra Water	F.A.*	C.A.**
M1	400	0	0	40	184	390	1234
M2	400	0	39	32	165	394	1247
M3	400	0	77	24	146	398	1259
M4	400	0	116	17	127	402	1272
M5	329	71	0	40	184	390	1234
M6	329	71	39	32	165	394	1247
M7	329	71	77	24	146	398	1259
M8	329	71	116	17	127	402	1272
M9	270	130	0	40	184	390	1234
M10	270	130	39	32	165	394	1247
M11	270	130	77	24	146	398	1259
M12	270	130	116	17	127	402	1272
M13	226	174	0	40	184	390	1234
M14	226	174	39	32	165	394	1247
M15	226	174	77	24	146	398	1259
M16	226	174	116	17	127	402	1272
M17	450	0	131	19	143	549	1019
M18	500	0	145	21	159	512	950
M19	550	0	160	23	175	475	881
M20	400	0	116	17	107	604	1122
M21	400	0	116	17	147	568	1054
M22	400	0	116	17	87	622	1156

*F.A., fine aggregate.

**C.A., coarse aggregate.

### Specimen preparation and testing

The mixing protocol in this study was as follows: The dry materials (GGBFS or GGBFS + QL, and aggregates) were first thoroughly mixed in the mixer pan for about a minute. Then, the pre-prepared alkaline activator was added and continued mixing for around 3 min till the total mix became homogenous. The alkaline activator was prepared by dissolving the SH flakes with potable water to get the SH solution, and then the SS solution was added to the prepared SH solution with good stirring until a homogenous solution was achieved; the activator solution was allowed to release the heat in sufficient time to reach a temperature of about 30–35 °C before adding to the dry materials in the mixing process.

The workability, in terms of initial slump value and slump loss with time, for all mixes was assessed. The slump test was conducted in accordance with ASTM C143^18^. The initial slump value was recorded just after mixing, while the rate of slump loss was observed by recording the slump value with time until losing the most of initial slump value.

In accordance with BS EN 12390-1^19^, the specimens were cast into steel molds of 100 × 100 × 100 mm to determine the concrete compressive strength. After 24 h of concrete casting, the specimens were removed from molds and cured in the lab at a temperature of 25 ± 2 °C until reaching the specified testing time. Compressive strength was reported for all mixes at the ages of 1st, 3rd, 7th, 14th, 28th, and 56th day, using three test specimens for each age, to investigate the development of compressive strength. The compression test was carried out according to BS EN 12390-3^20^.

## Test results and discussion

### Workability

#### Initial slump value

**Figure 3:**
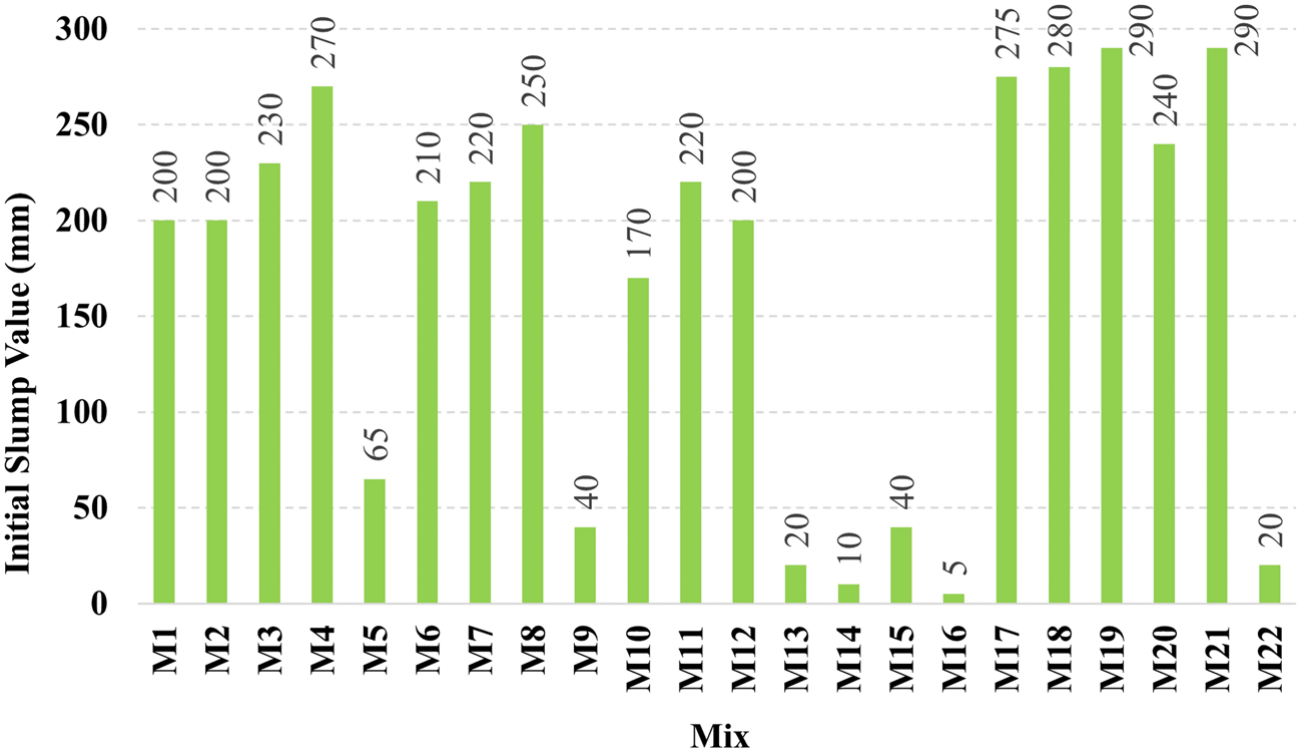
The initial slump value for all the mixes.

**Figure 4:**
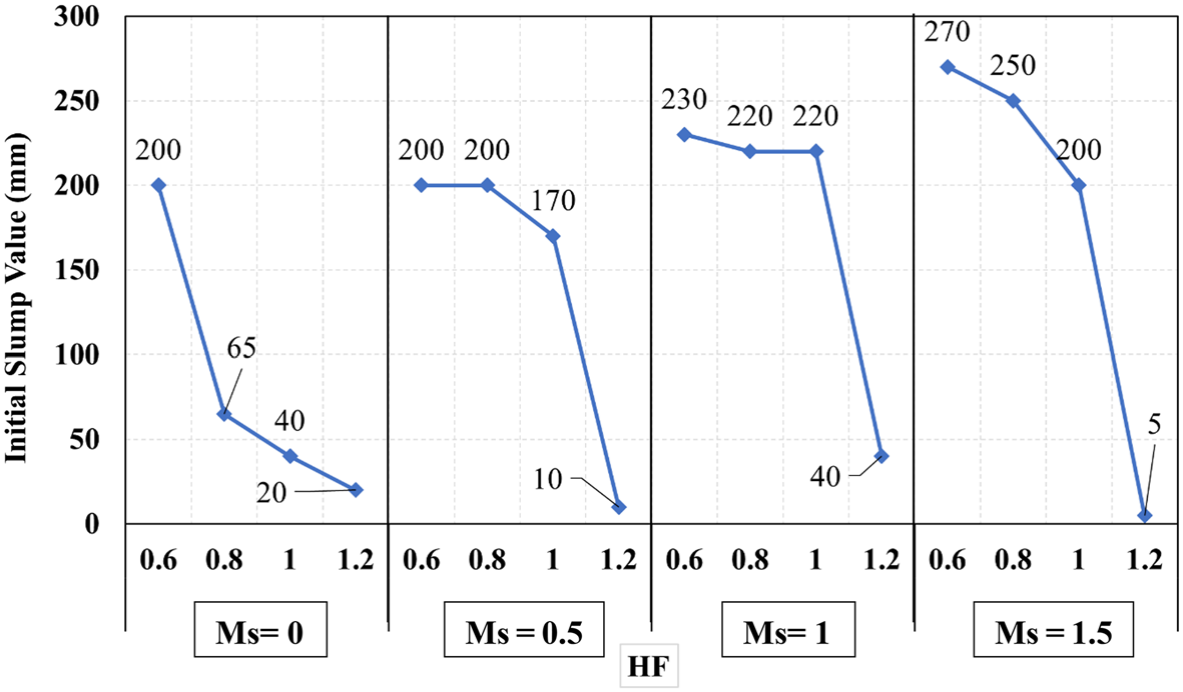
The relationship between initial slump values and HF values at the different levels of Ms.

**Figure 5:**
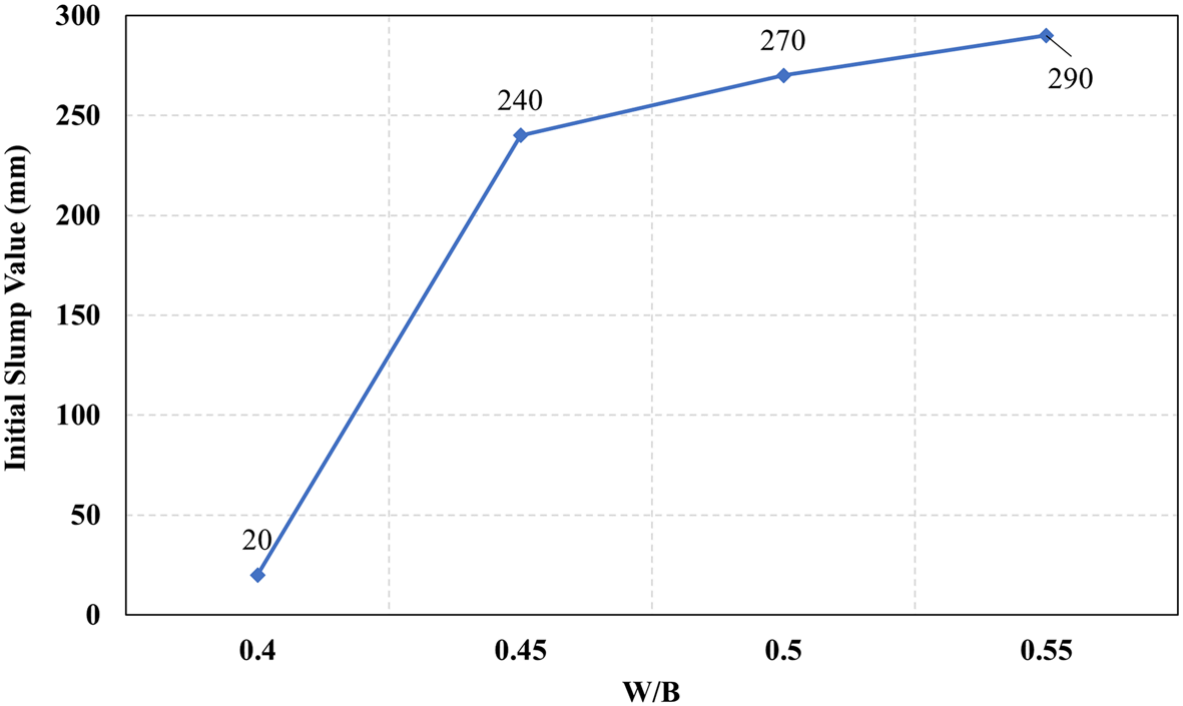
The relationship between initial slump values and W/B ratio.

**Figure 6:**
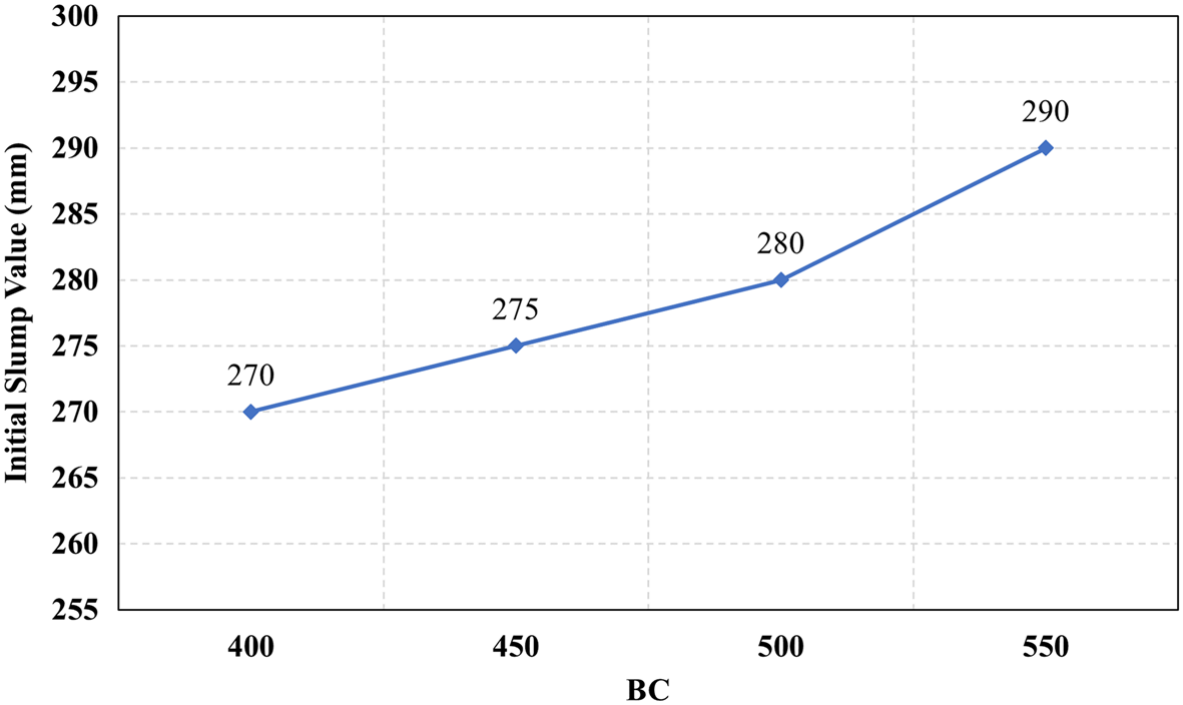
The relationship between initial slump values and Binde Content.

The recorded initial slump values of all mixes are presented in Fig. 3. It can be observed that mix M16 had achieved the lowest initial slump value, which can be attributed to the high CaO content which reacts rapidly in the presence of the high alkalinity activator to form CSH gel. In contrast, mix M4, M17, M18, M19, M20, and M21 achieved the highest initial slump values, which can be attributed to the low CaO content. From Fig. 3 it was observed that set 1 and set 2 have a clear trend as the slump increases as the Ms increases, while for set 3 a decrease in the slump was observed at Ms 1.5, and set 4 didn’t achieve any clear trend but slump lowered significantly this might be attributed to increased content of CaO, that react rapidly and adsorbs water fast while at the same time, the slag content reduced thus eventually the lubrication action reduced significantly leading to reduced slump. The relationship between the initial slump value and the HF value at different levels of Ms for all the studied mixes is illustrated in Fig. 4. It was observed in Fig. 4 that increasing the hybrid factor (HF) value results in decreasing the initial slump value whatever the solution modulus (Ms). This can be explained through the fact that the increase in the HF value increases the amount of Ca ions in the binder, which reacts rapidly in the presence of the activator faster than other oxides. These results coincide with previous research^10,13,21,22^. The relationship between the initial slump value and W/B ratio for all the studied mixes is illustrated in Fig. 5. The increase in the water-to-binder ratio has a positive effect on the initial slump value as the water acts as a lubricant to the dry materials. However, it was observed that if the water-to-binder ratio reduced below 0.45 the slump reduces crucially, this observation agrees with the previous literature^15,23^, this decrement might be due to the reduced free water that acts as a lubricant between mixture components. The relationship between the initial slump value and different levels of binder content (BC) for all the studied mixes is illustrated in Fig. 6. It can be found from Fig. 6 that increasing the binder content improves the initial slump value, which can be explained through the ratio of total liquids to binder content, to maintain the same W/B ratio of 0.5 and the Ms of 1.50 as the binder content increases the extra water, SH, and SS content increase thus the total liquids increase, the other reason that would justify this increment is the increased paste volume that acts as a lubricant to reduce the friction between aggregates thus increase the slump values.

#### Slump loss

**Figure 7:**
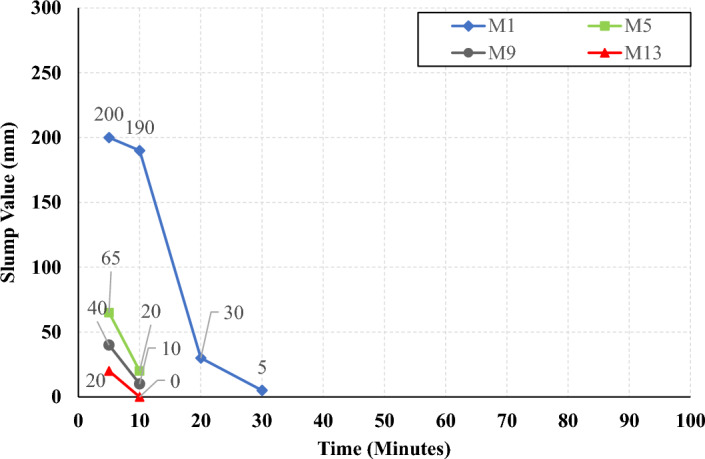
The slump values versus time for the different HF levels at Ms = 0.

**Figure 8:**
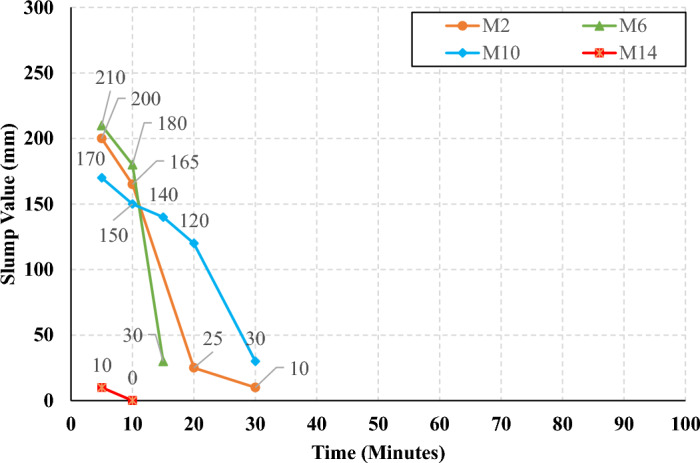
The slump values versus time for the different HF levels at Ms = 0.5.

**Figure 9:**
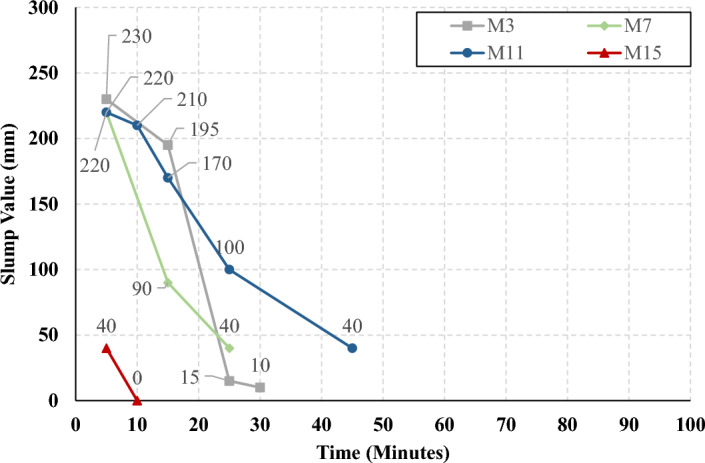
The slump values versus time for the different HF levels at Ms = 1.0.

**Figure 10:**
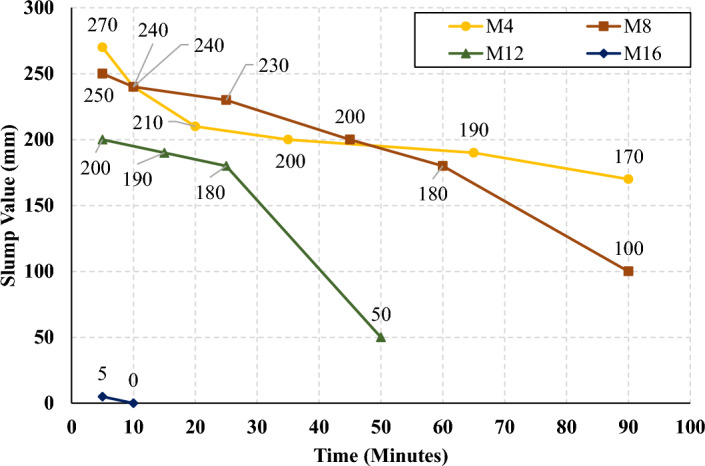
The slump values versus time for the different HF levels at Ms = 1.5.

**Figure 11:**
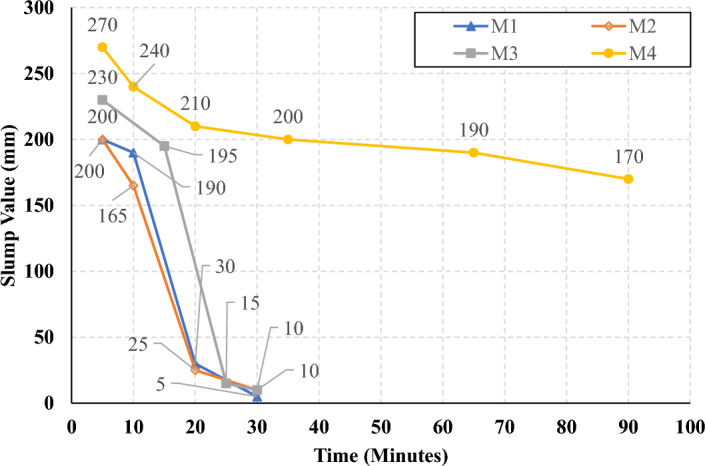
The slump values versus time for the different Ms levels at HF = 0.6.

**Figure 12:**
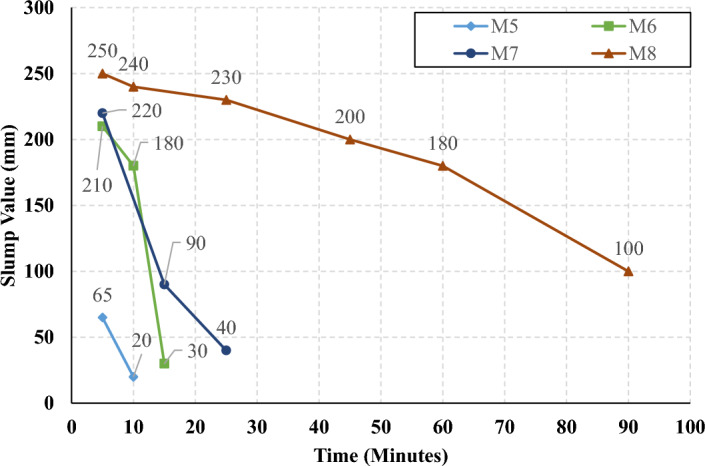
The slump values versus time for the different Ms levels at HF = 0.8.

**Figure 13:**
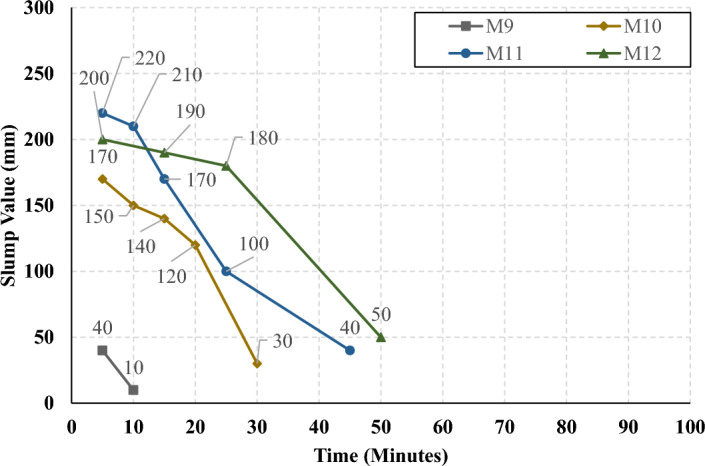
The slump values versus time for the different Ms levels at HF = 1.0.

**Figure 14:**
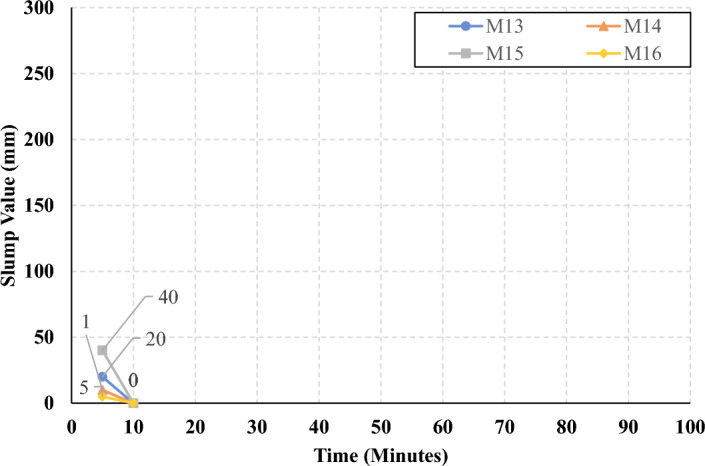
The slump values versus time for the different Ms levels at HF = 1.2.

The rate of slump loss was investigated in all studied mixes through the first 90 min just after mixing, the slump test was conducted under controlled environmental conditions, ensuring consistent temperature and relative humidity throughout the experimentation process. Figures 7, 8, 9, and 10 present the measured slump values versus time for the mixes that have different levels of HF and constant Ms of 0, 0.5, 1.0, and 1.5, respectively. It can be observed that the slump loss rate can be improved by increasing the Ms, which can be attributed to the increase of the Si ions. In addition, Figs. 11, 12, 13, and 14 present the measured slump values with time for the mixes that have different levels of Ms and constant HF of 0.6, 0.8, 1.0, and 1.2, respectively. It can be observed that increasing the HF value above 1.0 resulted in a high slump loss rate as clarified in Fig. 14, which can be attributed to the increased CaO content and the increased reaction degree of the CaO at this percentage of replacement (25% at HF = 1.2)^9^. Increasing the solution modulus (Ms) led to an improvement in the slump loss rate for the hybrid factors up to HF = 1, as clarified in Figs. 11, 12, and 13. This can be explained through the following: (i) Increasing the solution modulus (Ms) results in increasing the Si ions; (ii) The Si ions lower the alkalinity of the activator which results in prolonged time for the process of activation and sequentially lower slump loss rate, this finding agrees with the previous literature^24–27^.

### Compressive strength

**Figure 15:**
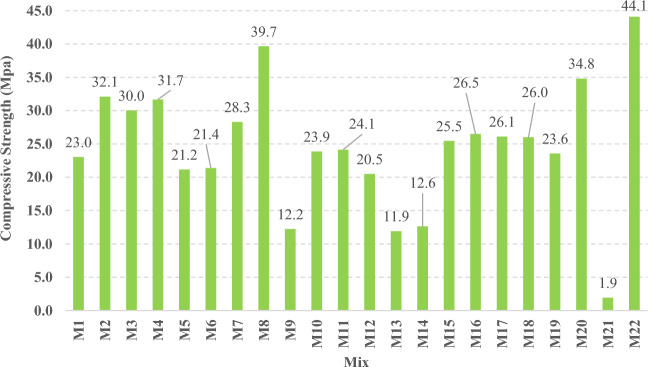
The compressive strength of all mixes at 56 days.

All mixes were tested in compression through ages of 1, 3, 7, 14, 28, and 56 days, three specimens were tested at each age and the average value was used in the results presentation and analysis. The recorded cubic compressive strength values at 56 days of all mixes are presented in Fig. 15. It can be observed that M22 had achieved the highest 56 days compressive strength, which can be attributed to the low W/B ratio. In contrast, M21 had achieved the lowest 56 days compressive strength which can be attributed to the high W/B ratio.

**Figure 16:**
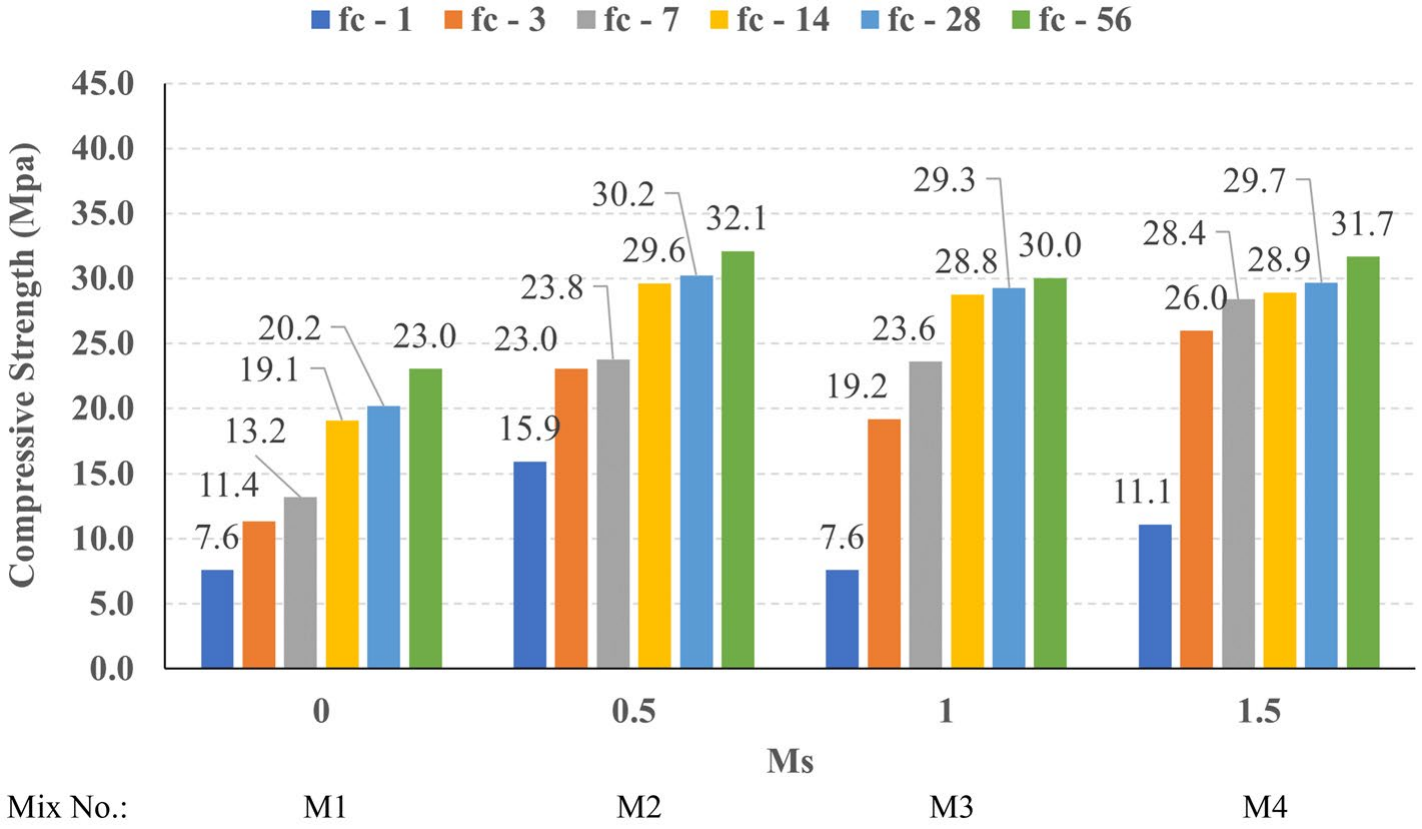
The compressive strength values versus time for the different Ms levels at HF = 0.60.

**Figure 17:**
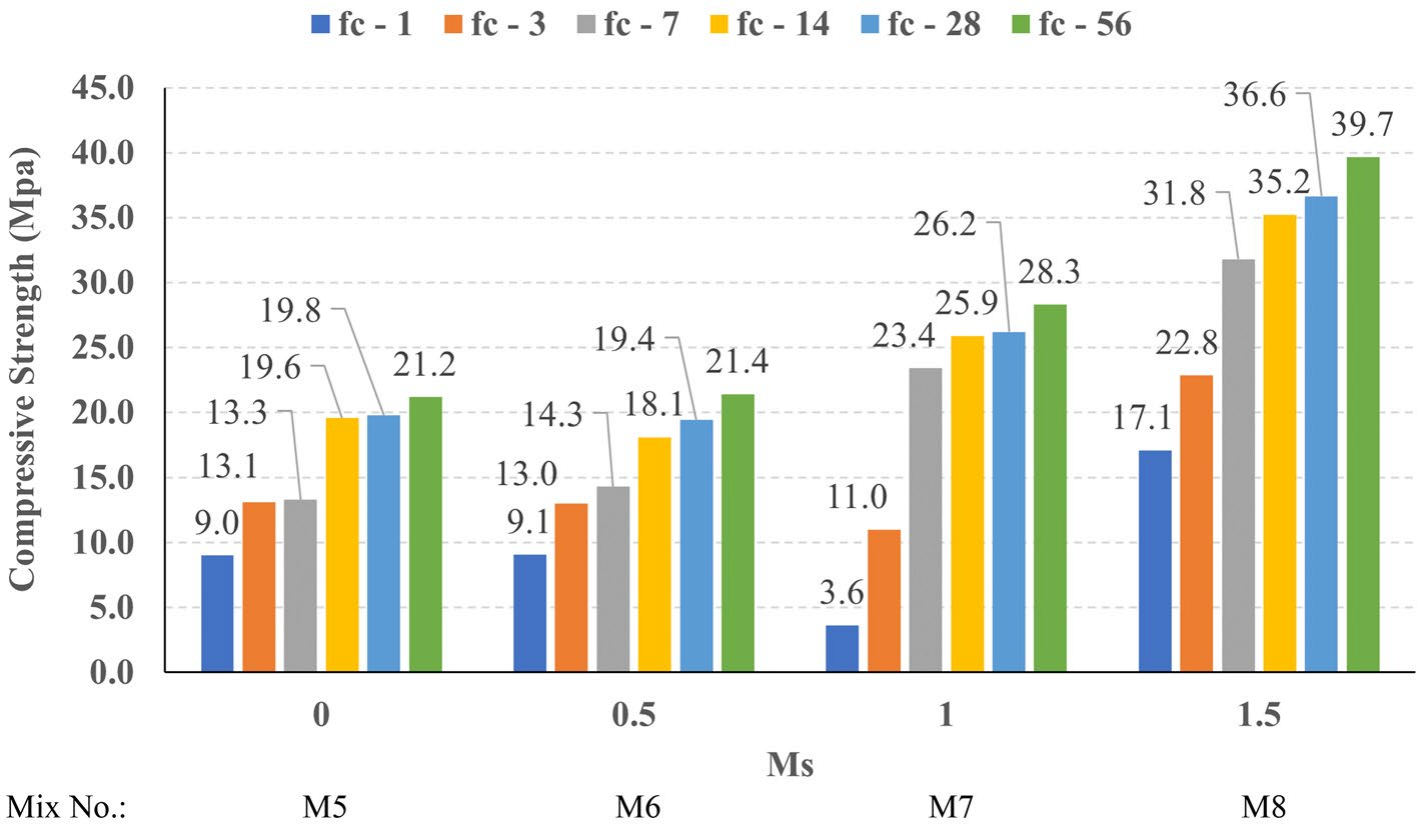
The compressive strength values versus time for the different Ms levels at HF = 0.80.

**Figure 18:**
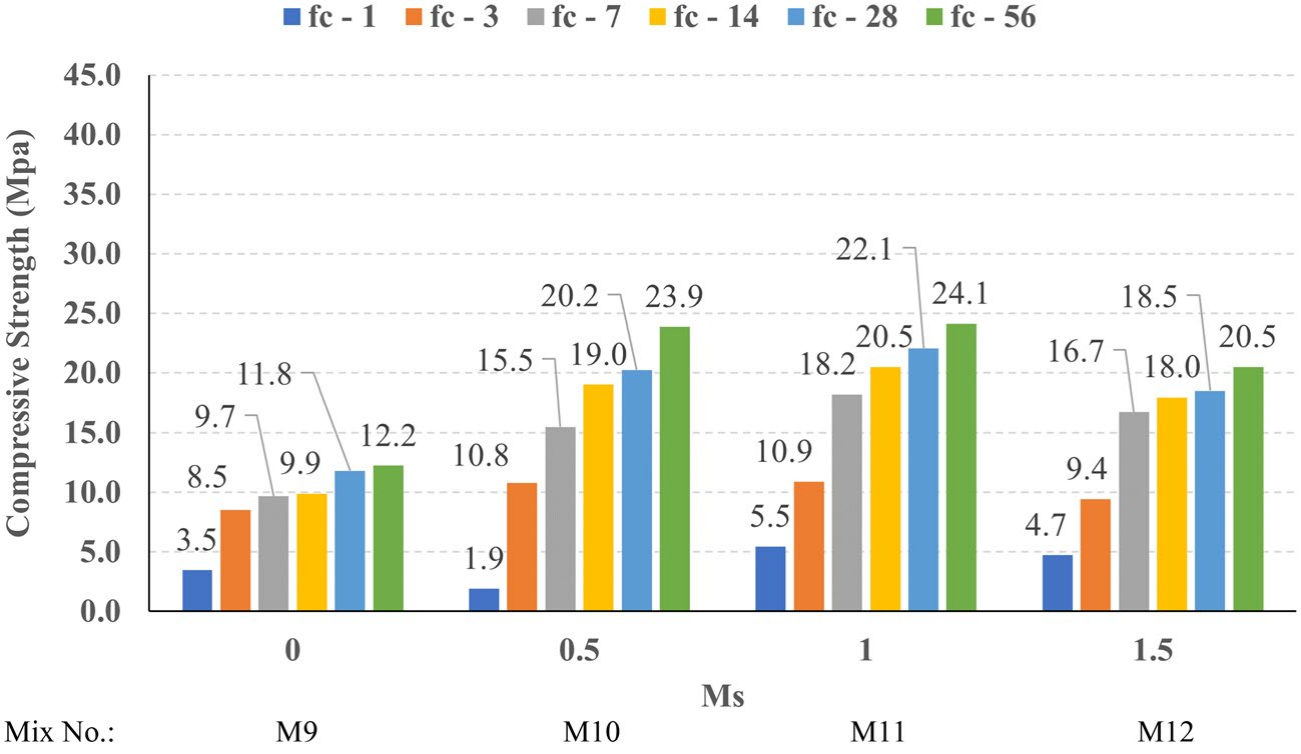
The compressive strength values versus time for the different Ms levels at HF = 1.00.

**Figure 19:**
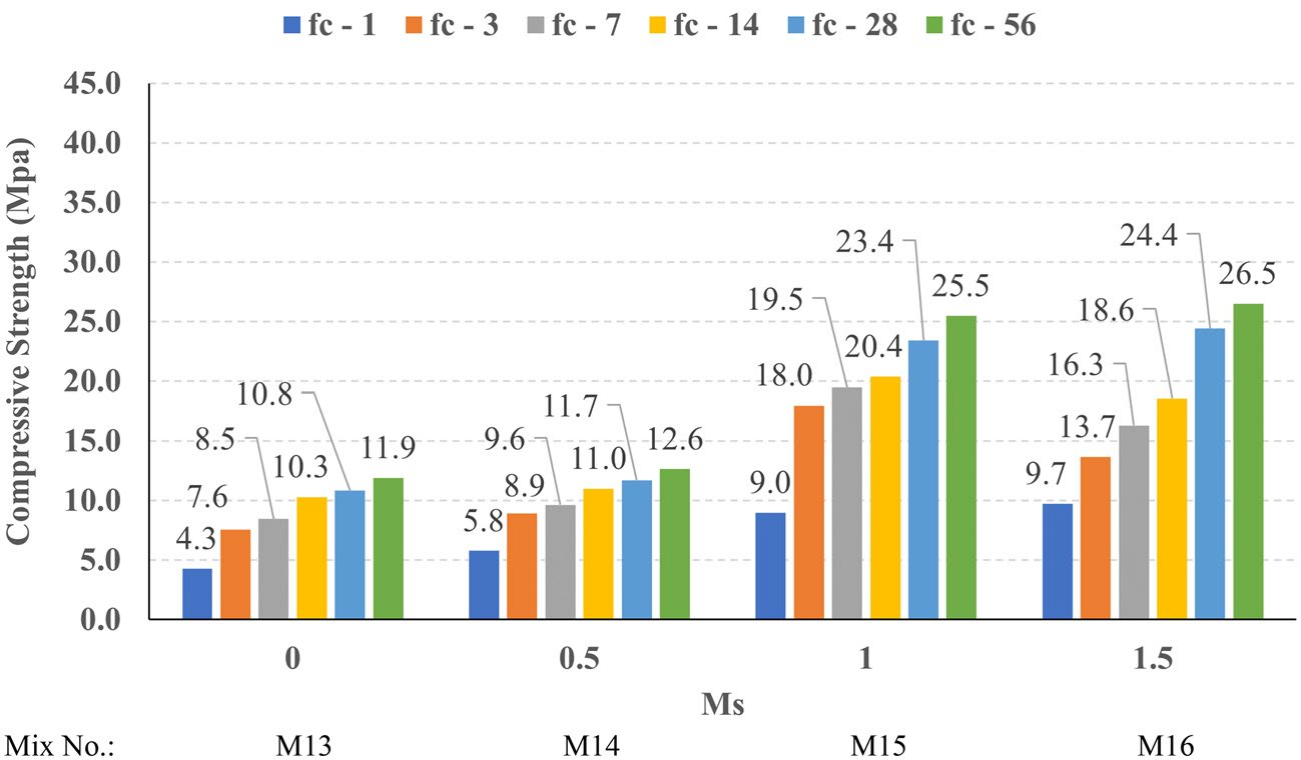
The compressive strength values versus time for the different Ms levels at HF = 1.20.

**Figure 20:**
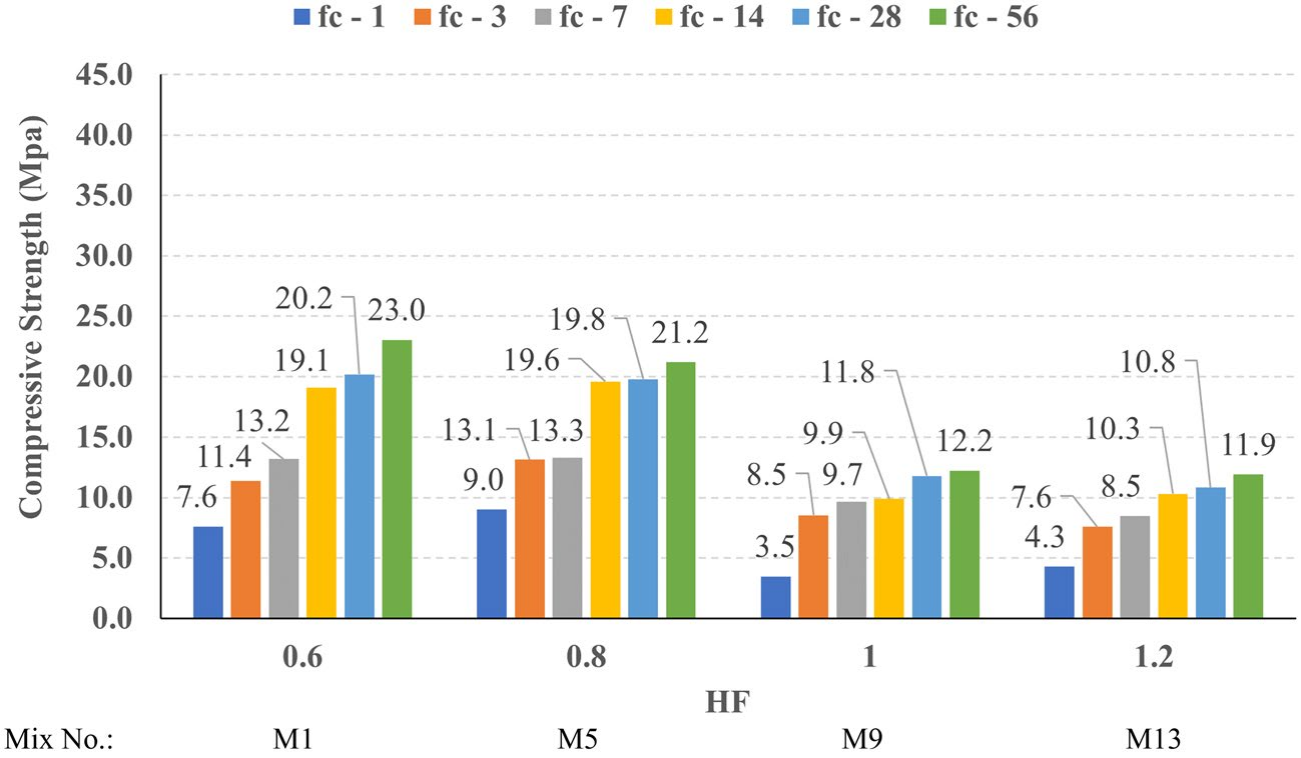
The compressive strength values versus time for the different HF levels at Ms = 0.0.

**Figure 21:**
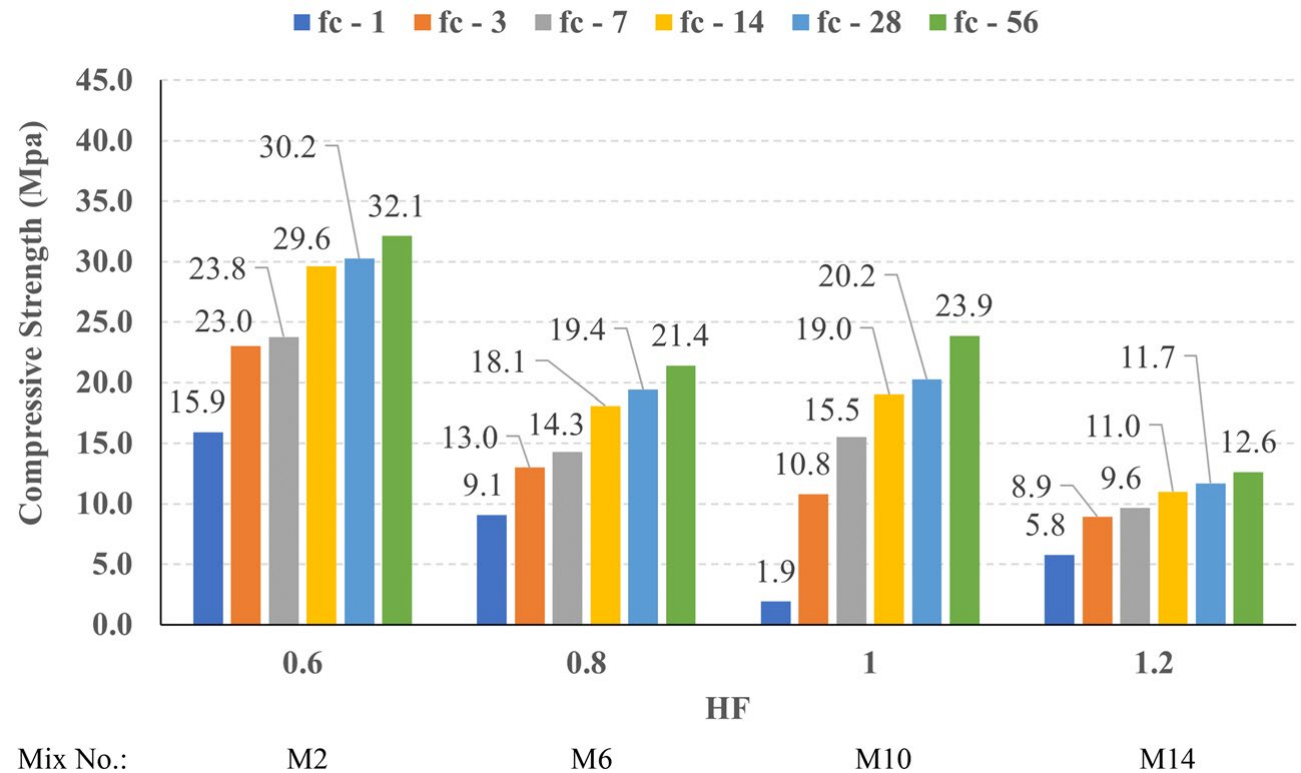
The compressive strength values versus time for the different HF levels at Ms = 0.5.

**Figure 22:**
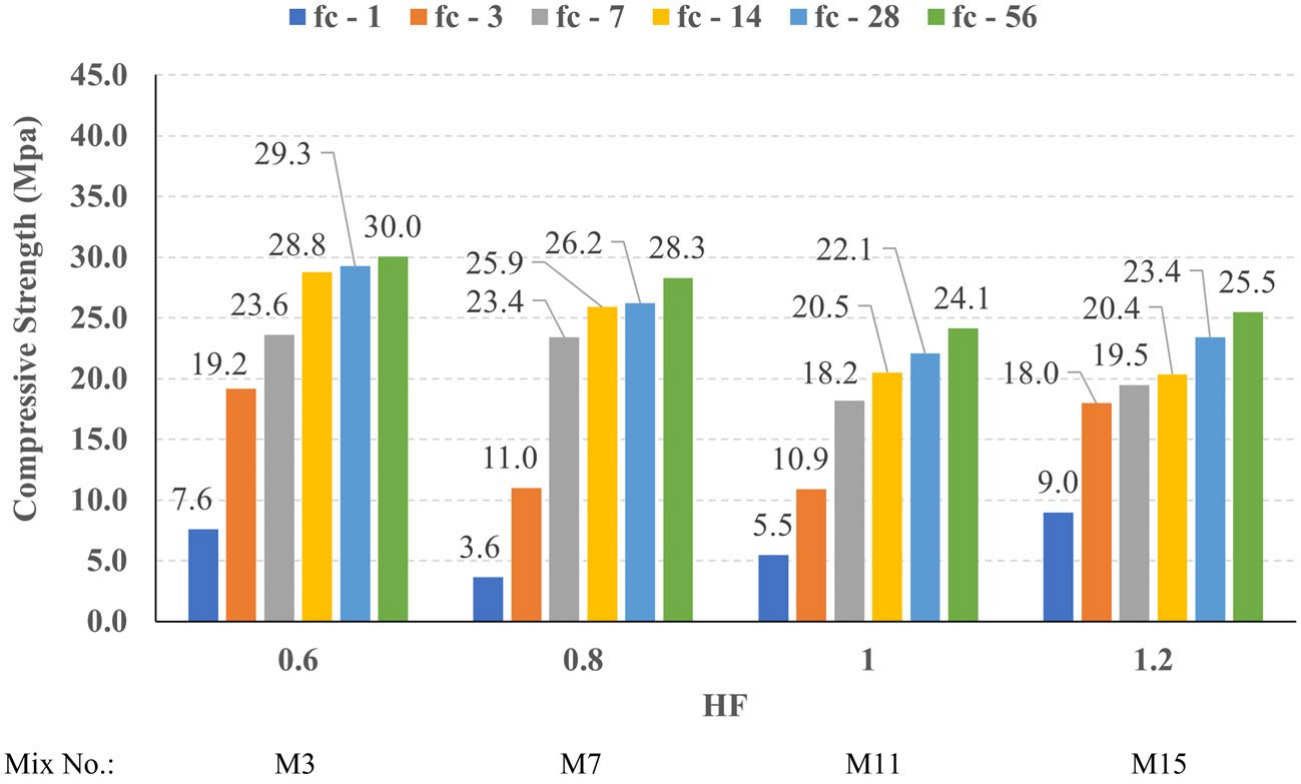
The compressive strength values versus time for the different HF levels at Ms = 1.0.

**Figure 23:**
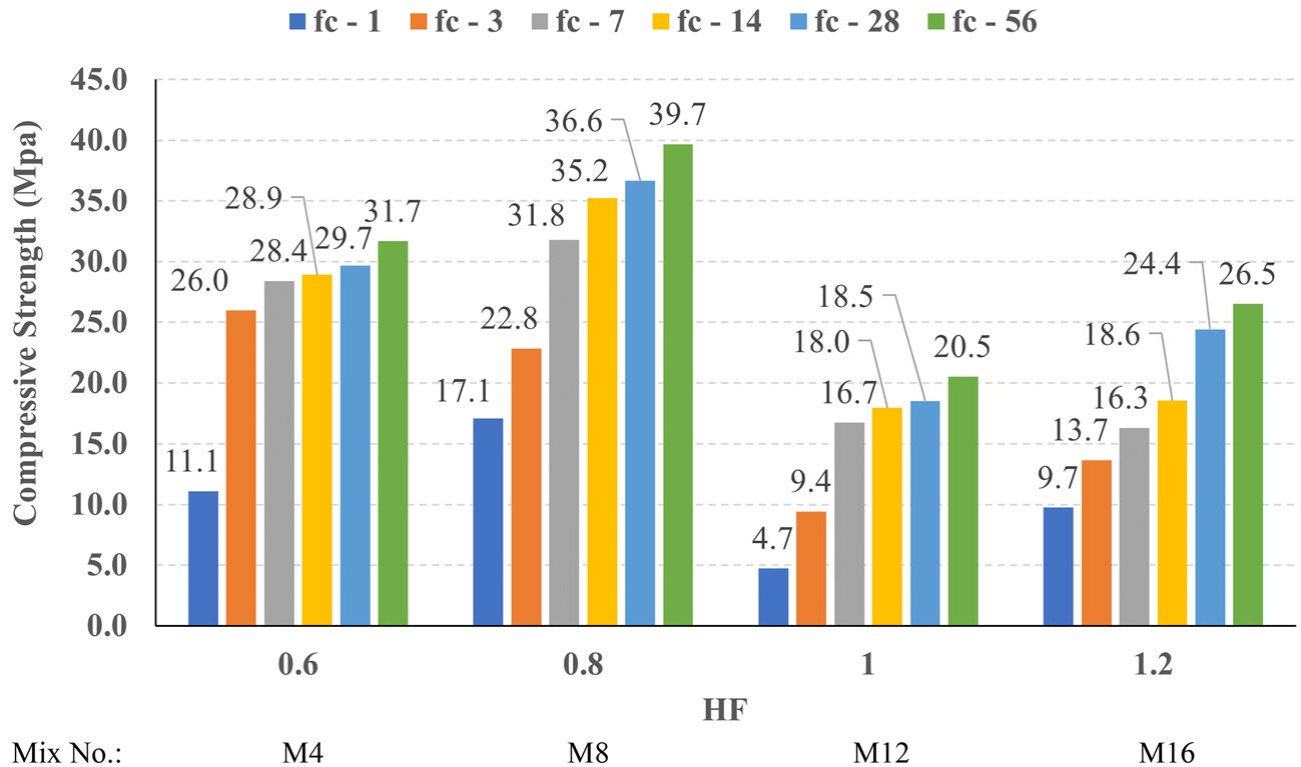
The compressive strength values versus time for the different HF levels at Ms = 1.5.

**Figure 24:**
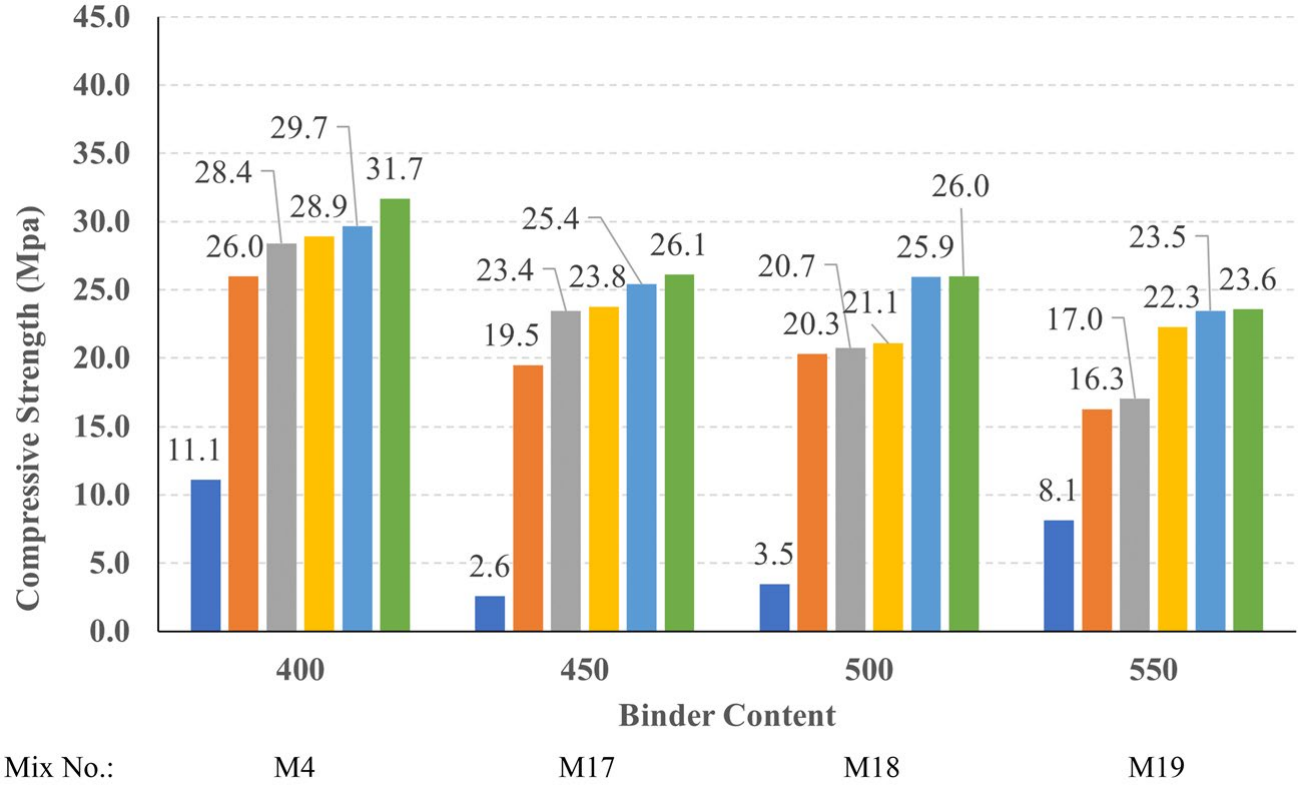
The compressive strength of different binder contents.

**Figure 25:**
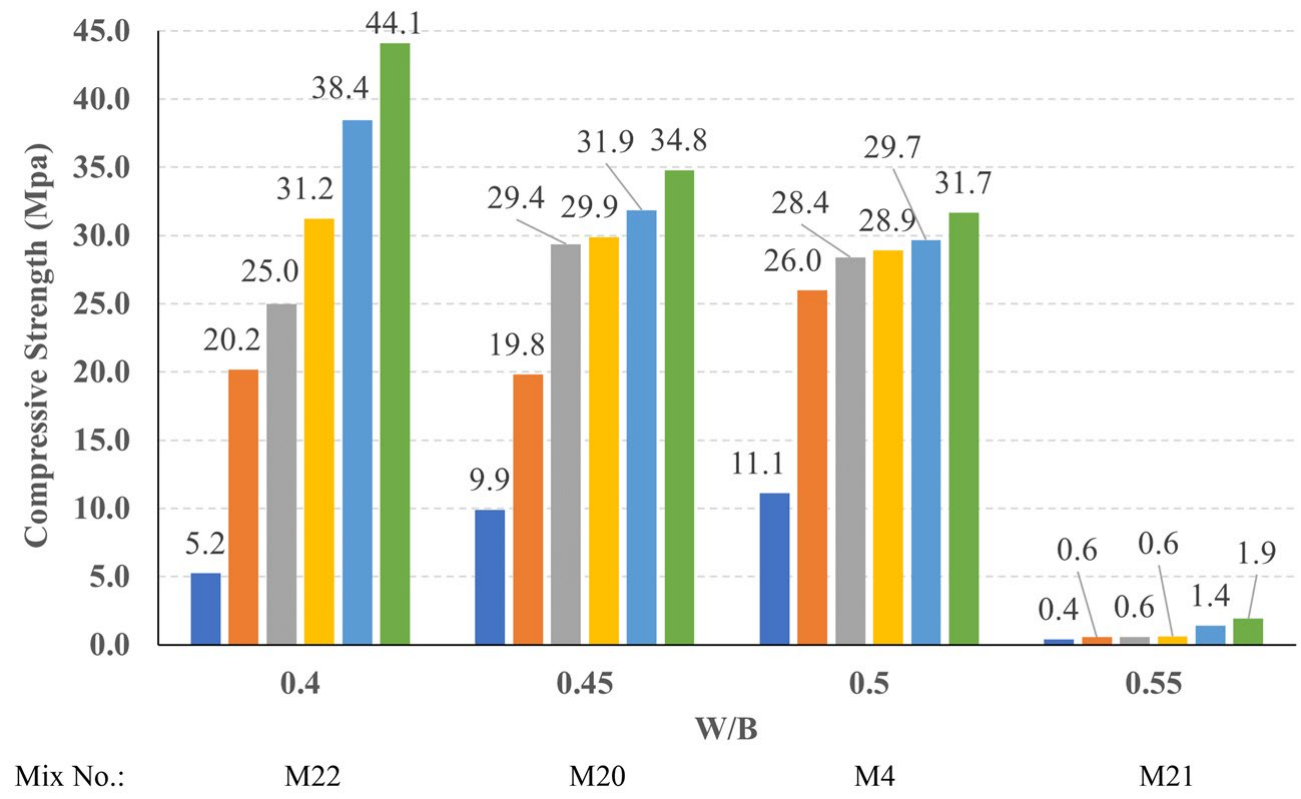
The compressive strength at different W/B levels.

**Figure 26:**
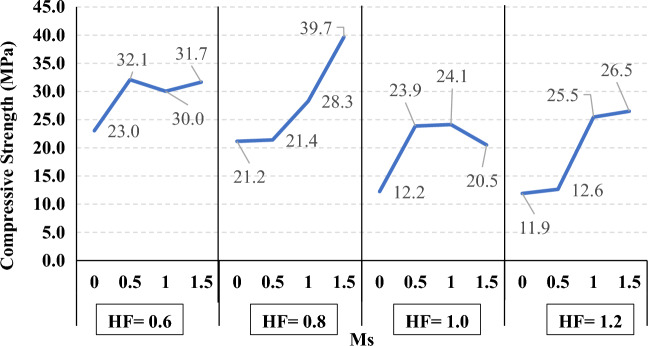
The relationship between the compressive strength and Ms values at the different levels of HF.

**Figure 27:**
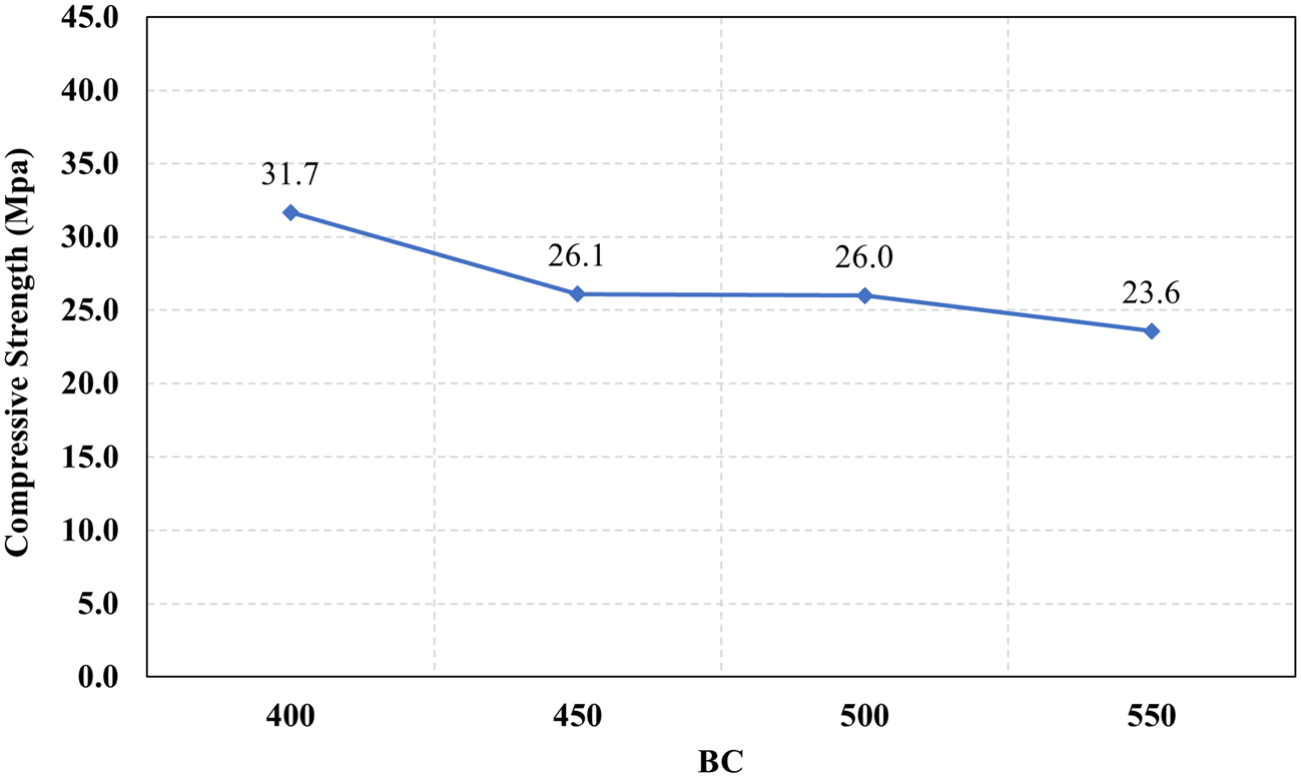
The relationship between the compressive strength and the different levels of BC.

**Figure 28:**
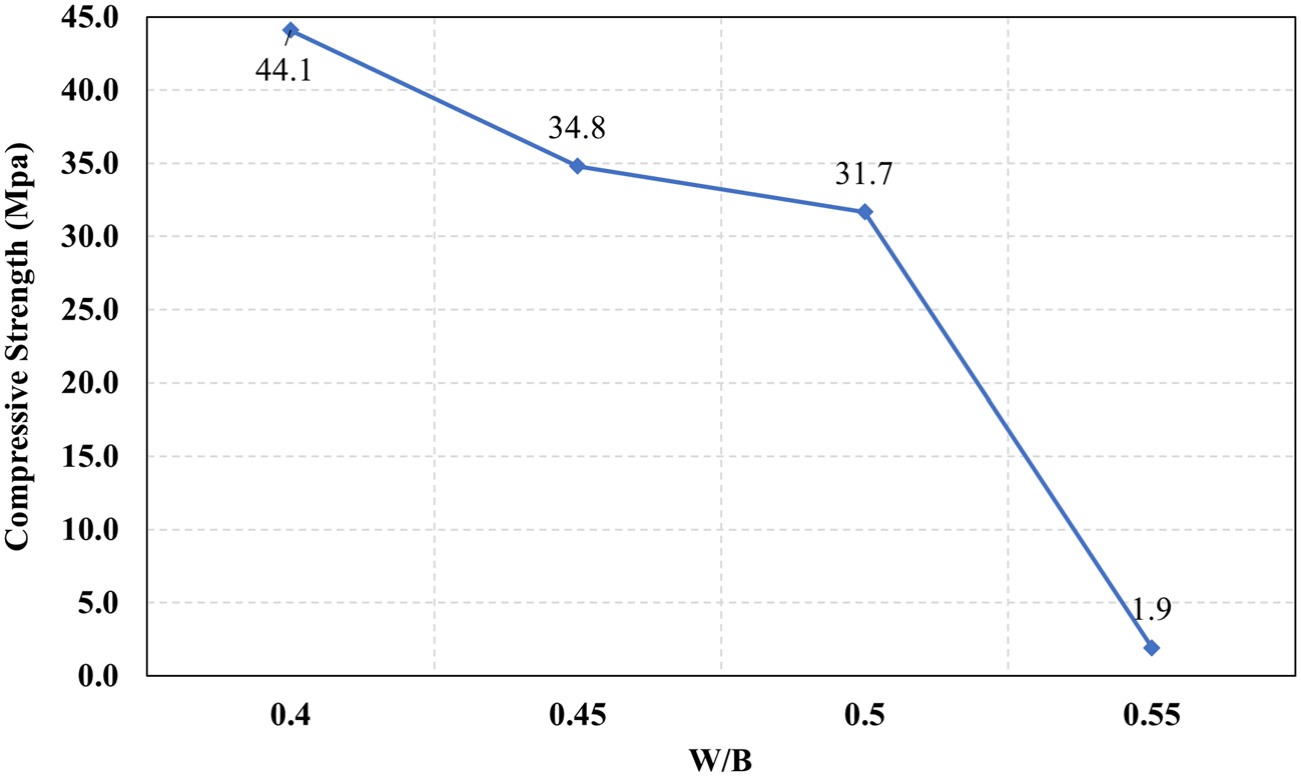
The relationship between the compressive strength and the different levels of W/B.

The compressive strength development through the first 56 days was investigated in all studied mixes. Figures 16, 17, 18, and 19 present the reported compressive strength values with time for the mixes that have different levels of Ms and constant HF of 0.6, 0.8, 1.0, and 1.2, respectively. Figures 20, 21, 22, and 23 present the reported compressive strength values versus time for the mixes that have different levels of HF and constant Ms of 0, 0.5, 1.0, and 1.5, respectively. Figure 24 presents the reported compressive strength values with time for the mixes that have different levels of binder content (BC) at the same HF of 0.6 and the same Ms of 1.5. Figure 25 presents the reported compressive strength values with time for the mixes that have different levels of water-to-binder ratio (W/B) at the same HF of 0.6 and the same Ms of 1.5. The relationship between the 56 days compressive strength and the Ms values at different levels of HF for all the studied mixes are illustrated in Fig. 26, the relationship between the 56 days compressive strength and the different levels of W/B ratio for all the studied mixes are illustrated in Fig. 27, and the relationship between the 56 days compressive strength and different levels of binder content (BC) for all the studied mixes are illustrated in Fig. 28. From Fig. 26 it can be concluded that the silicate-based (Ms > 0) activators achieve better compressive strength whatever the HF level, this can be attributed to the better microstructure and denser matrix of the formed matrix^27,28^.

At HF of 0.60, increasing the Ms above zero led to an improvement in the compressive strength but no obvious trend was noted while increasing the solution modulus value from 0.5 to 1.5. Further research is required to extend the solution modulus range above 1.5 to determine the trend at this level of the hybrid factor. At HF of 0.80 increasing the Ms led to better compressive strength which can be explained through the Ca/Si ratio, decreasing the Ca/Si ratio would lead to better compressive strength as the CSH and CASH phases increase and the molar volume decreases resulting in higher specific surface area and better cohesive forces^28,29^. At HF of 1.0 increasing the Ms above zero led to an improvement in the compressive strength but no obvious trend was noted while increasing the solution modulus value from 0.5 to 1.5. Further research is required to extend the solution modulus range above 1.5 to determine the trend at this level of the hybrid factor. At HF of 1.2 increasing the Ms level led to an improvement in the compressive strength although the worse workability, this might be explained through the hydration process of the added limestone as at a certain level of limestone addition (25% replacement by weight which achieves HF = 1.16) the hydration process improved and results in higher compressive strength^9^.

Generally, increasing the CaO content (HF level) would increase or reduce the compressive strength, at low replacement ratios (Low HF) CaO boosts the slag dissolution degree and improves the microstructure of AAS thus the compressive strength increases, while at high replacement ratios (High HF) CaO reduces the compressive strength due to the dilution action of the reduced slag content and coarsen the microstructure^30^ and formation of macropores in the binding paste^31^. Also, the low HF levels (0.6 and 0.8) achieved better slump, and slump loss, consequentially better compaction which also may explain the better compressive strength compared to high HF levels (1.0 and 1.2). From Figs. 24 and 27 it can be concluded that increasing the binder content would reduce the compressive strength which can be explained through that increasing binder content leads to increased total liquids to the binder, this increment in liquids would increase the drying shrinkage which result in increased cracks hence a reduced compressive strength. From Figs. 25 and 28 it can be concluded that increasing the W/B ratio up to 0.50 would reduce the compressive strength which can be explained through that increasing the W/B ratio means increasing the free Si ions which reduces the alkalinity of the activator and hence the activation degree, also increasing the free water would result eventually in increasing the voids in the microstructure. At the W/B ratio of 0.55, almost the AASC didn’t react as at a certain level of free Si ions and free water (W/B = 0.55) the alkalinity reduced so severely that the slag was not activated^28^.

## Conclusions

Based on the analysis and discussion of the experimental program test results of this research, the following can be obtained:

1. Increasing the hybrid factor (CaO content) would reduce the workability of the AAS concrete disregarding the solution modulus value.
2. Increasing the solution modulus up to a certain level would reduce the alkalinity of the activator relatively thus prolonging the activation time and improving the workability.
3. Like ordinary concrete, increasing the water-to-binder ratio achieves better workability but reduces the compressive strength.
4. Increasing the binder content while maintaining the same W/B ratio would achieve lower compressive strength due to the increment of total liquids to binder ratio which worsens the concrete compressive strength due to the increased shrinkage and voids.
5. The silicate-based activators lead to better fresh and hardened state properties compared to sodium hydroxide activators.
6. The effective range to achieve desirable workability and concrete compressive strength is from HF 0.6 up to 0.8 at Ms 1.5. This would achieve a compressive strength of more than 30 MPa and a slump of 100 mm after 90 min.

## Data Availability

The datasets used and/or analyzed during the current study are available from the corresponding author upon reasonable request.

## References

[1] Costa FN, Ribeiro DV (2020). Reduction in CO_2_ emissions during production of cement, with partial replacement of traditional raw materials by civil construction waste (CCW). J. Clean. Prod..

[2] Mohamad N, Muthusamy K, Embong R, Kusbiantoro A, Hashim MH (2021). Environmental impact of cement production and Solutions: A review. Mater. Today Proc..

[3] Zhao J (2021). Eco-friendly geopolymer materials: A review of performance improvement, potential application and sustainability assessment. J. Clean. Prod..

[4] Farooq F (2021). Geopolymer concrete as sustainable material: A state of the art review. Constr. Build. Mater..

[5] Provis JL, van Deventer JSJ (2014). Alkali materials activated state-of-the-art report. RILEM State-of-the-Art Rep..

[6] Zhou X, Zeng Y, Chen P, Jiao Z, Zheng W (2021). Mechanical properties of basalt and polypropylene fibre-reinforced alkali-activated slag concrete. Constr. Build. Mater..

[7] Jiao Z, Wang Y, Zheng W, Huang W (2018). Effect of dosage of sodium carbonate on the strength and drying shrinkage of sodium hydroxide based alkali-activated slag paste. Constr. Build. Mater..

[8] Jin Y (2020). Study on the interaction mechanism between slags and alkali silicate activators: A hydration kinetics approach. Constr. Build. Mater..

[9] Zhu X (2021). Chemical and physical effects of high-volume limestone powder on sodium silicate-activated slag cement (AASC). Constr. Build. Mater..

[10] Amer I, Kohail M, El-Feky MS, Rashad A, Khalaf MA (2021). Characterization of alkali-activated hybrid slag/cement concrete. Ain Shams Eng. J..

[11] Gebregziabiher BS, Thomas R, Peethamparan S (2015). Very early-age reaction kinetics and microstructural development in alkali-activated slag. Cem. Concr. Compos..

[12] Li N, Shi C, Zhang Z (2019). Understanding the roles of activators towards setting and hardening control of alkali-activated slag cement. Compos. B Eng..

[13] Cao R, Zhang S, Banthia N, Zhang Y, Zhang Z (2020). Interpreting the early-age reaction process of alkali-activated slag by using combined embedded ultrasonic measurement, thermal analysis, XRD, FTIR and SEM. Compos B Eng.

[14] Serag Faried A, Sofi WH, Taha AZ, El-Yamani MA, Tawfik TA (2020). Mix design proposed for geopolymer concrete mixtures based on ground granulated blast furnace slag. Aust. J. Civ. Eng..

[15] Li N (2018). A mixture proportioning method for the development of performance-based alkali-activated slag-based concrete. Cem. Concr. Compos..

[16] Gunasekara, M. P. C. M., Law, D. W. & Setunge, S. Effect of composition of raw materials on compressive strength of fly ash based geopolymer mortar. In *Proceedings of the 27th Biennial National Conference of the Concrete Institute of Australia* Vol. 23**,** 586–595 (Concrete Institute of Australia, 2015).

[17] ASTM Standards C33/C33M - 16. *Designation: C33/C33M − 16´116´1 Standard Specification for Concrete Aggregates 1*. *ASTM Standards C33/C33M* **04.02** (2016).

[18] ASTM Standard C134/C134M-15a. *Standard Test Method for Slump of Hydraulic-Cement Concrete C143/C143M − 15a*. (2015). 10.1520/C0143_C0143M-15A

[19] EN British Standard 12390-1. *Testing hardened concrete Part 1: Shape, dimensions and other requirements for specimens and moulds* (2012).

[20] EN British Standard 12390-3. *Testing hardened concrete–Part 3: Compressive strength of test specimens* (British Standard Institution, 2002).

[21] Fernandez-Jimenez A, Puertas F (2001). Setting of alkali-activated slag cement. Influence of activator nature. Adv. Cem. Res..

[22] Krizan D, Zivanovic B (2002). Effects of dosage and modulus of water glass on early hydration of alkali-slag cements. Cem. Concr. Res..

[23] Hung CC, Chang JJ (2013). The influence of mixture variables for the alkali-activated slag concrete on the properties of concrete. J. Mar. Sci. Technol..

[24] Gebregziabiher BS, Thomas RJ, Peethamparan S (2016). Temperature and activator effect on early-age reaction kinetics of alkali-activated slag binders. Constr. Build. Mater..

[25] Fernandez-Jimenez A, Puertas F (2003). Effect of activator mix on the hydration and strength behaviour of alkali-activated slag cements. Adv. Cem. Res..

[26] Huanhai Z, Xuequan W, Zhongzi X, Mingshu T (1993). Kinetic study on hydration of alkali-activated slag. Cem. Concr. Res..

[27] Bílek V, Novotný R, Koplík J, Kadlec M, Kalina L (2023). Philosophy of rational mixture proportioning of alkali-activated materials validated by the hydration kinetics of alkali-activated slag and its microstructure. Cem. Concr. Res..

[28] Shi Z, Shi C, Wan S, Li N, Zhang Z (2018). Effect of alkali dosage and silicate modulus on carbonation of alkali-activated slag mortars. Cem. Concr. Res..

[29] Kunther W, Ferreiro S, Skibsted J (2017). Influence of the Ca/Si ratio on the compressive strength of cementitious calcium-silicate-hydrate binders. J. Mater. Chem. A Mater..

[30] Lin Chan C, Zhang M (2023). Effect of limestone on engineering properties of alkali-activated concrete: A review. Constr. Build. Mater..

[31] Liu X, Li B, Chen YT, Shi W, Ghiassi B (2023). Role of limestone powder in alkali-activated slag paste with superabsorbent polymer. J. Build. Eng..

